# Machine Learning and Simulation-Optimization Coupling for Water Distribution Network Contamination Source Detection

**DOI:** 10.3390/s21041157

**Published:** 2021-02-06

**Authors:** Luka Grbčić, Lado Kranjčević, Siniša Družeta

**Affiliations:** 1 Department of Fluid Mechanics and Computational Engineering, Faculty of Engineering, University of Rijeka, 51000 Rijeka, Croatia; lado.kranjcevic@riteh.hr (L.K.); sinisa.druzeta@riteh.hr (S.D.); 2 Center for Advanced Computing and Modelling, University of Rijeka, 51000 Rijeka, Croatia

**Keywords:** random forests, water network contamination, simulation-optimization, machine learning, pollution source identification, fireworks algorithm, MADS

## Abstract

This paper presents and explores a novel methodology for solving the problem of a water distribution network contamination event, which includes determining the exact source of contamination, the contamination start and end times and the injected contaminant concentration. The methodology is based on coupling a machine learning algorithm for predicting the most probable contamination sources in a water distribution network with an optimization algorithm for determining the values of contamination start time, end time and injected contaminant concentration for each predicted node separately. Two slightly different algorithmic frameworks were constructed which are based on the mentioned methodology. Both algorithmic frameworks utilize the Random Forest algorithm for classification of top source contamination node candidates, with one of the frameworks directly using the stochastic fireworks optimization algorithm to determine the contamination start time, end time and injected contaminant concentration for each predicted node separately. The second framework uses the Random Forest algorithm for an additional regression prediction of each top node’s start time, end time and contaminant concentration and is then coupled with the deterministic global search optimization algorithm MADS. Both a small sized (92 potential sources) network with perfect sensor measurements and a medium sized (865 potential sources) benchmark network with fuzzy sensor measurements were used to explore the proposed frameworks. Both algorithmic frameworks perform well and show robustness in determining the true source node, start and end times and contaminant concentration, with the second framework being extremely efficient on the fuzzy sensor measurement benchmark network.

## 1. Introduction

Functional water supply networks are essential for a proper urban environment and the population that inhabits it. Monitoring the quality of water in the water supply network, and in case of contamination, identifying and controlling the source and contamination propagation is an extremely important task for human health and safety. Water supply network pollution can be caused by a wide variety of incidents which include an intentional contamination, biofilm formation in pipes, water aging and chemical contamination from pipe lining and corrosion [1,2].

Water supply network security methodologies heavily rely on accurate water quality models and pipe network hydraulic simulators. EPANET [3] is the most popular simulator created for the purposes of running simulation experiments which are therefore used, in conjunction with various mathematical methodologies, for finding the optimal water quality sensor placement in a water supply network ([4,5,6,7]), control of water supply networks in case of contamination events ([8,9,10]) and contamination source detection ([11,12,13]). A thorough and recent review of methodologies for water supply network quality modeling with contamination source detection can be found in [14] and a general, recent thorough review of water supply network security research and methods are covered in [15].

Simulation-optimization methods have been the most popular approach for the water supply network contamination source detection problem. This procedure includes the coupling of an optimization algorithm (stochastic or deterministic) with a water supply network simulator. The goal function of the optimization algorithm is to minimize the difference between the recorded water quality sensor readings and the simulated values in order to find the contamination source, start and end times of the contamination event and the injected concentration of the contaminant. Genetic algorithms (GA) and variations have been widely used for this purpose ([16,17,18]). Simulation-optimization with added hydraulic demand uncertainty and GA has also been investigated [19]. Recently, a Poisson model for a changing water demand was coupled with an improved GA [20].

The simulation-optimization approach comes with an added computational cost and is usually parallelized due to the fact that the problem variables are both of discrete (network nodes) and continuous nature (contamination start and end times, injected contaminant concentration). Beside the GA stochastic approach, the Nelder-Mead (NM) deterministic optimization algorithm was used coupled with logistic regression to determine the potential contamination source candidates and other relevant variables [21]. An important feature of this work is that it proposed a model-based approach for classifying the most probable contamination source nodes and thus eliminated the discrete variable from the simulation-optimization procedure and applied it only to the other relevant variables for the contamination event reconstruction. Recently, an algorithm for search space reduction was developed for eliminating potential source nodes based on a sensor measurement comparison procedure [22]. The simulation-optimization approach was then applied for the remaining potential source nodes. Both Particle Swarm Optimization (PSO) and GA were investigated and PSO exhibited better convergence rate and accuracy. Other simulation-optimization based methods include dynamic niching GA [23], cultural algorithm [24], hybrid encoding [25] and a data-driven multi-strategy collaboration algorithm [26].

Another approach to solving the problem of source identification is to use Bayesian optimization. In the work by [27] a Bayesian framework for localizing multiple pollution sources and it incorporated Gaussian process emulators trained on data obtained from computational fluid dynamics simulations. A Bayesian approach was investigated for the contamination source localization in a water distribution network with stochastic demands [28], and recently, reference [29] constructed a Bayesian framework for the same application of contamination source localization but with mobile sensor data. Additionally, a Gaussian surrogate model was implemented with a collaborative based algorithm [30] specifically for the contamination source identification problem.

Recently, machine learning methods have been successfully applied to a wide variety of problems in environmental engineering. A Long Short-Term Memory (LSTM) Neural Network was used for the problem of flood forecasting with rainfall and discharge as input data [31]. Additionally, Artificial Neural Networks (ANN) and Random Forests (RF) were coupled to identify chemical leaks using data obtained from monitoring [32]. Similarly to air quality prediction, the field of groundwater flow modeling has also been actively including machine learning methods. Convolutional Neural Network (CNN) coupled with a Markov Chain Monte Carlo (MCMC) method has been used to identify the contaminant sources in groundwater flow [33].

Alternatively, it is possible to use machine learning algorithms for contamination source detection in water supply networks. Artificial Neural Network (ANN) was trained to detect the source of pollution of E. Coli in a small pipe network [34]. Potential sub-zones of contamination source nodes have been predicted using learning vector quantization Neural Network (LVQNN) for larger water supply pipe networks [35]. Recently, CNN has been used for the contamination source detection problem [36]. The CNN was trained based on the water supply network user complaints unlike the usual supply network water quality sensor recordings. Additionally, it was found that CNN performs better than a basic ANN. Recent work also includes a machine learning-based framework designed specifically for high performance systems [37]. The algorithmic framework uses ANNs for tournament style classification of potential contamination event source nodes and the Random Forest (RF) machine learning algorithm for regression analysis which predicts the contamination start and end times and injected contaminant concentrations.

Previously, Decision Trees (DT) were utilized for water network contamination source area isolation [38] and more recently, the RF algorithm has also been successfully utilized for potential water supply network contamination source node identification [39] and for determining the number of contamination sources in a water distribution network [40]. The RF algorithm was trained with Monte Carlo (MC) generated input data of sensor water quality readings through a time interval and the true source nodes as the output data. RF models were also trained with simulation data for the purpose of contamination source detection in river systems [41].

Machine learning and simulation-optimization coupling has been also employed in the area of groundwater pollution source and pollution characteristics prediction. Coupling of non-dominated sorting genetic algorithm II (NSGA-II) and both Probabilistic Support Vector Machines (PSVM) and Probabilistic Neural Networks (PNN) has been done for characterizing an unknown pollution source in groundwater resources systems [42].

In this work, a novel methodology for predicting the water supply network contamination event is presented and investigated. Two algorithmic frameworks are constructed which are based on the methodology. Both frameworks utilize a machine learning approach based on the RF algorithm (implemented in the Python machine learning module scikit-learn 0.21.3 [43]) for potential contamination source search space reduction (as presented in our previous work [39]). The first investigated framework couples the simulation-optimization procedure directly with the RF classifier in order to determine the contamination start time, end time and injected contaminant concentration for each RF model predicted node separately and for this framework, three different stochastic optimization algorithms were investigated for one water distribution network benchmark. The three stochastic optimization algorithms were Particle Swarm Optimization (PSO), fireworks algorithm (FWA), both implemented in the swarm optimization Python module indago 0.1.2 [44], and genetic algorithms (GA) implemented in the multiobjective optimization python module pymoo 0.4.2 [45]. The optimization algorithms were fine-tuned and the best performing one was further investigated with the coupling framework for both benchmark networks. The other algorithmic framework differs slightly as it includes an additional RF model regression for each RF predicted potential source node separately in order to predict each top node’s start time, end time and injected contaminant concentration. After the RF regression, each potential source node’s newly obtained data is then used as initial values for the deterministic global search optimization algorithm Mesh Adaptive Direct Search (MADS) which is implemented in NOMAD 4.0 [46].

The EPANET2 [3] hydraulic and water quality simulator is used for water supply network contamination event simulations. EPANET2 simulates contaminant transport using simplified complete mixing advection models which in most cases are not accurate enough as previously shown by [47]. However, for the purposes of examining the algorithm proposed in this study the simplified EPANET2 complete mixing model is good enough as the whole procedure is not dependent on the accuracy of the mixing processes occurring in the water distribution network. Monte Carlo simulations are made to train the RF model for classification (as described in [39]) with the sensor water quality measurements being the input features and the true source node being the output. The RF model classifier then predicts the most probable contamination source nodes which are then submitted either to the stochastic simulation-optimization procedure (for the first framework) or to RF regression (trained with previously generated MC EPANET2 data) which predicts their start and end times and injected contaminant concentration. Both algorithmic frameworks are used on two water supply benchmark networks. The smaller benchmark network (92 nodes) was investigated with perfect sensor water quality measurements, while the bigger (865 nodes) was investigated with fuzzy sensor water quality measurements.

## 2. Materials and Methods

### 2.1. Water Distribution Network Benchmarks

**Figure 1:**
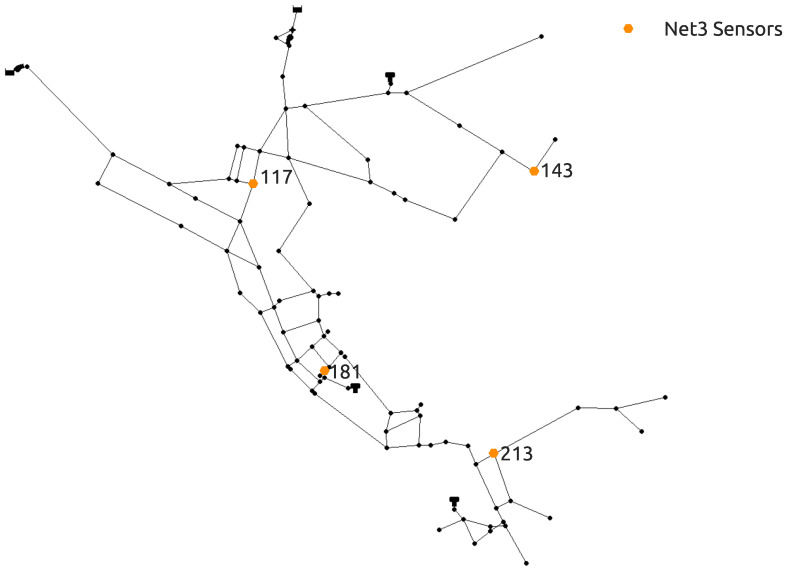
NET3 water distribution network benchmark with sensor positioning.

**Figure 2:**
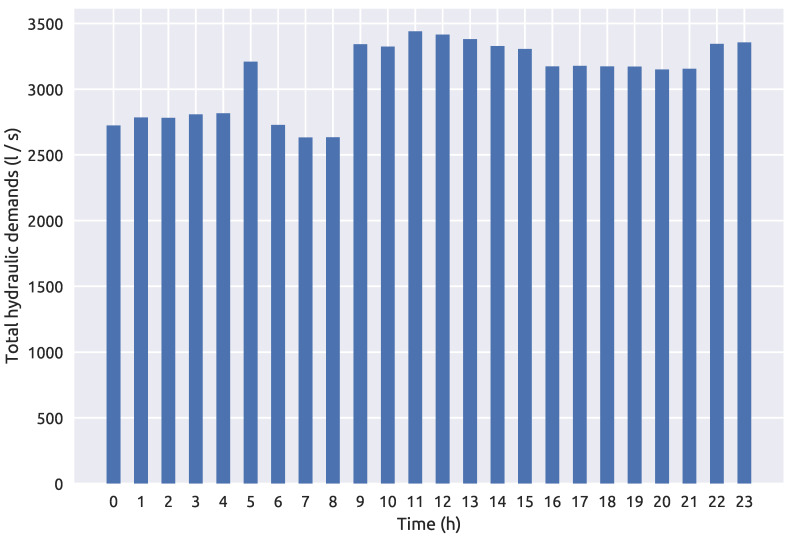
NET3 water distribution network total hydraulic demands over a 24 h period.

The novel methodology was tested on two benchmark water distribution networks. The first benchmark is the NET3 EPANET2 water distribution network consisting of 92 total nodes which are all initially considered as potential source nodes. A 24 h period simulation time was set with a 1 h time step for the hydraulic analysis and a 5 min time step for the water quality analysis. A contamination pattern was set with a 10 min time step with all network sensors recording the quality of water every hour through the whole simulation (0–24 h). The injected contaminant is chemically or biologically unspecified and is treated as a mass which is introduced into the water distribution network since the investigated frameworks are independent of the transport model used in the simulation. The sensor positioning was the same as the one introduced in [48] and which showed in [39] to include a good number of suspect nodes when used in conjunction with the RF algorithm for classification. The NET3 water distribution network with the selected sensor layout can be seen in Figure 1. Since the total number of sensors is 4, a total of 100 water quality recordings were made through one simulation as each sensor detects the water quality in the network each hour for the 24 h interval (including the initial water quality at 00:00 h). The total hydraulic load or total demands of all nodes of the NET3 benchmark network through a 24 h simulation time interval can be observed in Figure 2.

**Figure 3:**
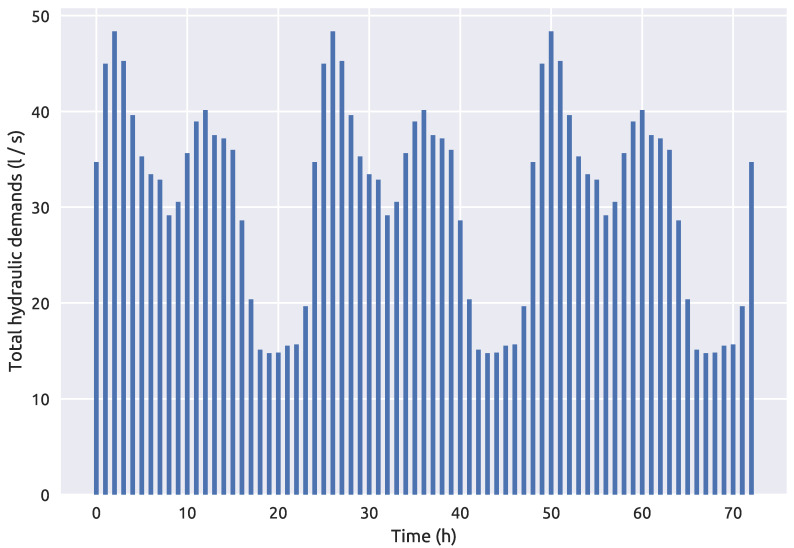
Richmond water distribution network total hydraulic demands over a 72 h period.

**Figure 4:**
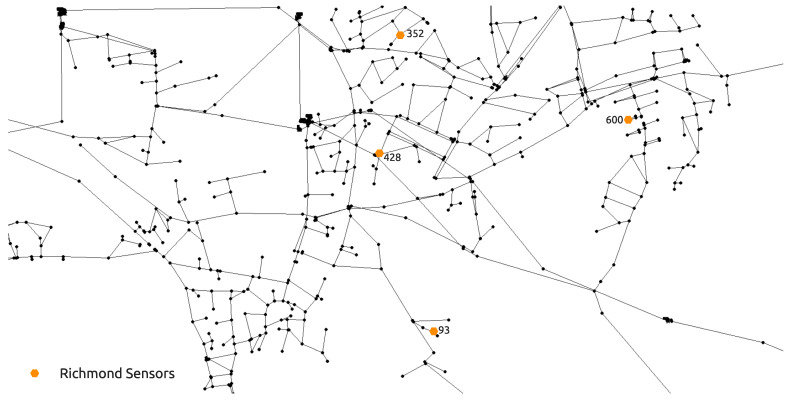
Richmond water distribution network benchmark detail with the positions of 4 sensors.

The second, larger water distribution network used for the purposes of examining the proposed methodology is the hydraulically calibrated Richmond water distribution network introduced by [49] and it was downloaded from the Centre for Water Systems [50] benchmark repository. This benchmark water distribution network is located in Yorkshire, UK and covers an area which is 14 km wide and 3 km long. The larger network consists of 865 potential source nodes. For this case, the simulation time interval was 72 h and a hydraulic analysis time step of 1 h, water quality time step of 5 min and a contamination pattern step of 1 h which was only set in the first 24 h of the simulation. The sensor positioning proposed by [16] was used as it performs well with the RF classifier as shown in [39]. The total node demands of the NET3 benchmark network through a 72 h simulation time interval can be observed in Figure 3. The detail of the Richmond water distribution network is seen in Figure 4 which includes the positioning of 4 sensors, while the fifth sensor is located at node 672 (which is not seen in Figure 4 as it is outside the shown detail).

All five sensors of the Richmond network recorded the quality of water every hour for the period of 72 h which entails a total of 365 measurements (73 per sensor). Additionally, the sensor measurements were set as fuzzy (categorical) and not perfect as in the NET3 benchmark. This means that the measurements were not the true physical value of the contaminant but only a value which defines if the contamination is low, medium or high. If the measured injected contaminant concentration of the contaminant *c* was 0<c<100 mg/L, the measured value was defined as low or 1; if it was 100≤c<500 mg/L, it was defined as medium or 2; and if it was c≥500 mg/L, the value was considered high or 3. The fuzzy sensor measurements are used to investigate the algorithmic frameworks for a lower quality sensor technology.

### 2.2. Machine Learning and Simulation-Optimization Coupling Framework 1

The first algorithmic framework consists of machine learning classification of potential contamination source nodes which is then coupled with simulation-optimization procedure for each node separately in order to determine the contamination event start time $s_{t}$ (h), end time $e_{t}$ (h) and injected contaminant concentration *c* (mg/L) by minimizing the difference between the measured water quality sensor readings and the simulated water quality sensor readings with changing the initial conditions ($s_{t}$, $e_{t}$ and *c*) of the simulated contamination scenario.

Firstly, the machine learning model classifier was built the same way as in the work by [39] using Random Forests. The input variables for training the ML model were water supply network’s sensor measurements through a time interval ($S_{0}\left(t_{0}\ldots t_{x}\right),S_{1}\left(t_{0}\ldots t_{x}\right),\ldots ,S_{n}\left(t_{0}\ldots t_{x}\right)$, where $S_{n}$ is the *n*-th sensor in the network and $t_{x}$ is the water quality measurement at time step *x*) while the output was the true contamination source node for each sensor measurement. All data for the RF model training was generated with Monte Carlo EPANET2 hydraulic and water quality analysis where the source node *N*, $s_{t}$, $e_{t}$ and *c* were varied. If a water supply network contamination event were to occur, the sensor measurements would be submitted to the trained RF model classifier and a list of top potential source nodes would be generated.

**Figure 5:**
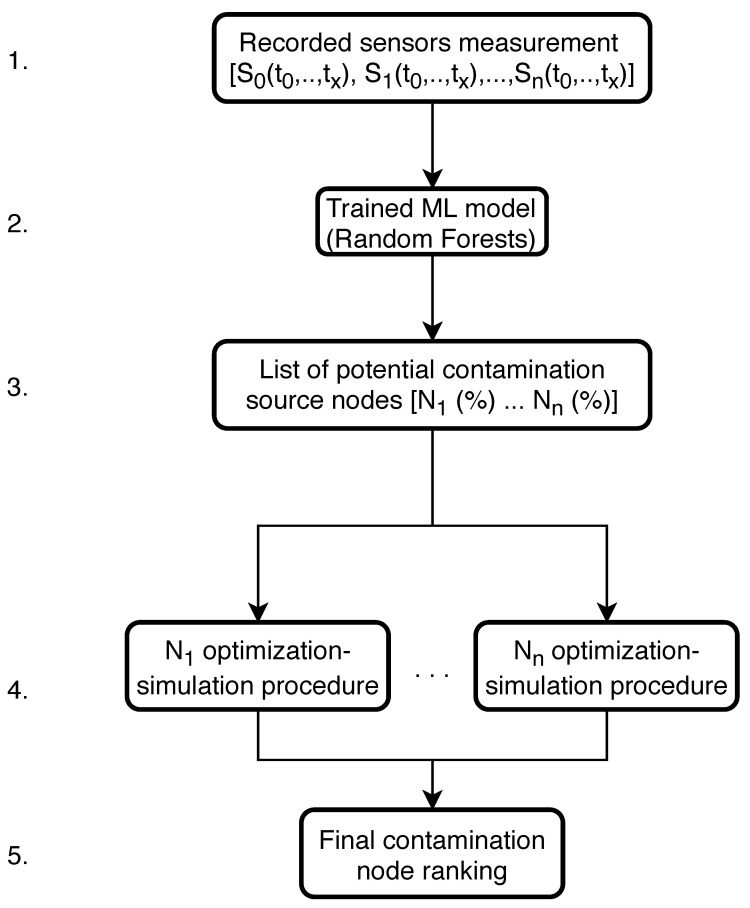
Machine learning and simulation-optimization coupling algorithmic framework 1 flowchart.

The whole algorithmic framework of machine learning and simulation-optimization coupling can be observed in Figure 5. The procedure of water supply network contamination event reconstruction starts with inputting the recorded sensors measurements time series into the trained machine learning model (marked as 1. in Figure 5). The trained RF model (step 2.) generates a prediction of the most probable contamination source nodes based on the water supply network sensor measurements time series and compiles them in a list where each predicted node has a corresponding individual probability (%) of being the true contamination source node (step 3.).

The next step (4.) of the algorithmic framework is to separately submit each predicted potential source node to the simulation-optimization procedure. The optimization variables are $s_{t}$, $e_{t}$ and *c* and the goal function *f* of the simulation-optimization procedure is defined as:

(1) $$f\left(s_{t},e_{t},c\right)=\sum_{i=0}^{n}\sum_{t=1}^{T}{\left(c_{i}^{s}\left(t\right)-c_{i}^{m}\left(t\right)\right)}^{2}$$

where *n* is the number of water supply network sensors, *T* the simulation duration with a time step *t*, $c^{m}$ is the measured injected contaminant contaminant from the real contamination event and $c^{s}$ represents the simulated values of the contaminant. For the exact solution the goal function must yield zero for the true contamination source node.

When the simulation-optimization procedure is finished for each node, a final contamination node ranking is obtained (step 5.). The node with the smallest value of *f* (Equation (1)) can be considered the true contamination event source node. A problem can arise with the final source node ranking due to the strong multi-modal nature of the problem, which means that several potential source nodes can simultaneously converge to the same minimum value of *f*. The best performing stochastic optimization algorithm described in Section 2.5 was used for coupling with the ML classification.

### 2.3. Machine Learning and Simulation-Optimization Coupling Framework 2

**Figure 6:**
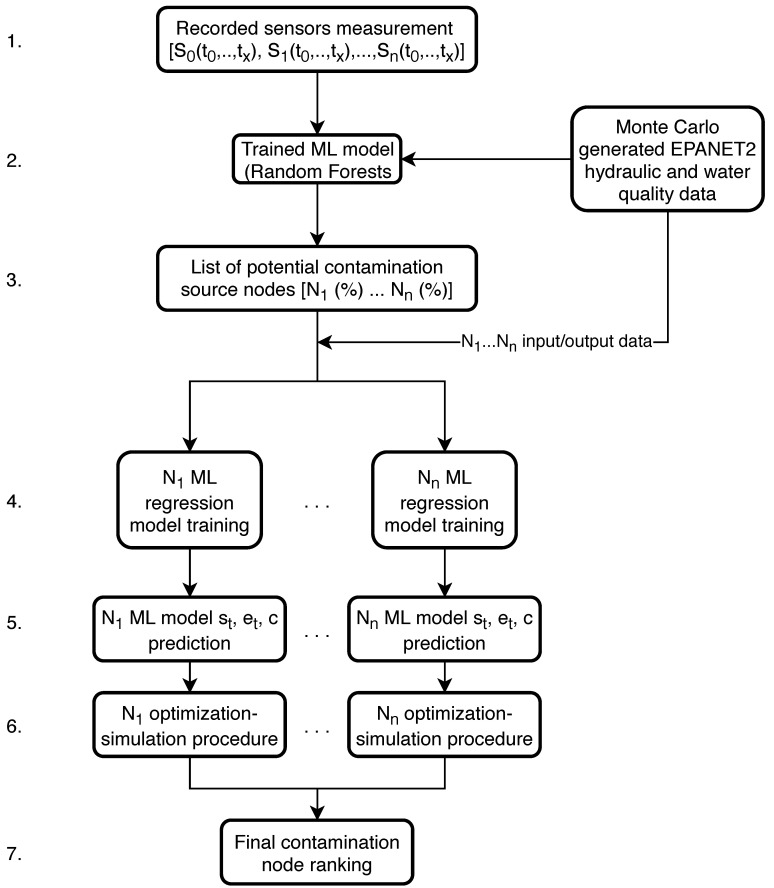
Machine learning and simulation-optimization coupling algorithmic framework 2 flowchart.

This coupling framework differs slightly from the one described in Section 2.2. The general procedure is the same with an additional machine learning model regression added before the optimization algorithm with a to determine the values of $s_{t}$, $e_{t}$ and *c* using ML algorithms. Figure 6 shows the coupling framework.

**Figure 7:**
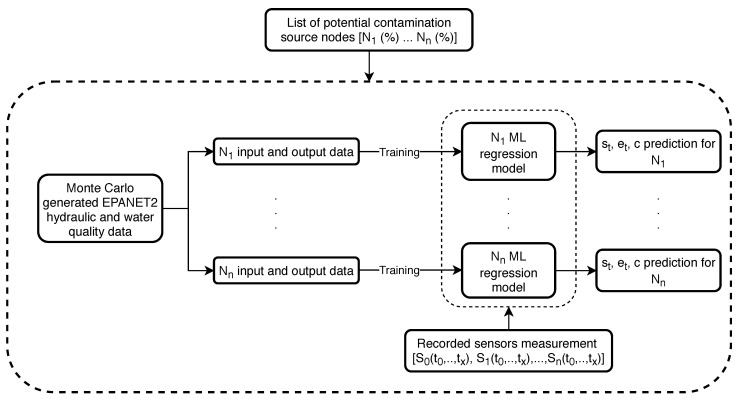
Machine learning regression model flowchart.

Step 4. of the algorithmic framework is to separately train ML regression models for each predicted potential contamination source node (from steps 1.–3.) using pre-generated Monte Carlo data (EPANET2 hydraulic and water quality analysis) which was also used for the ML model classifier training (step 2.). This step is done in parallel where each node’s ML regression model is trained on a separate CPU. The input data of each node’s ML regression model training were the water supply network sensor measurements and the outputs were the corresponding values of $s_{t}$, $e_{t}$ and *c*. After each node’s ML regression models were trained, the recorded sensor measurements (from step 1.) were then used for prediction of $s_{t}$, $e_{t}$ and *c* (step 5.). Steps 4. and 5. of the algorithmic framework can be observed in Figure 7 with more detail.

Previously generated data by Monte Carlo simulations (which is also used to build the general ML classifier from step 2. in Figure 6 is used to build an individual node’s regression model. The input data for each node *N* are the simulated sensor readings ($S_{0}\left(t_{0}\ldots t_{x}\right),S_{1}\left(t_{0}\ldots t_{x}\right),\ldots ,S_{n}\left(t_{0}\ldots t_{x}\right)$) while the output data are the values of $s_{t}$, $e_{t}$ and *c* of each corresponding sensor reading. After the ML models are trained for all potential nodes (with all of the input and output data), the initial recorded sensor measurements are used as data inputs to generate each node’s predictions of $s_{t}$, $e_{t}$ and *c*. The Random Forest algorithm is also used for the machine learning regression in Step 4. as it was shown to work well for contamination source variables regression in the work by [37].

After each potential source node obtains a prediction of $s_{t}$, $e_{t}$ and *c* based on the recorded sensor measurements, the predicted values are passed to the next step (6.) of the framework. This step utilizes the simulation-optimization procedure for each node separately which entails that the only left optimization variables are continuous. Each node’s predicted values of $s_{t}$, $e_{t}$ and *c* are used as initial search values for a simulation-optimization procedure which utilizes a deterministic optimization algorithm. The goal function *f* of the simulation-optimization procedure is defined in Equation (1). The deterministic global search algorithm Mesh Adaptive Direct Search (MADS) was used in this coupling procedure and is described in Section 2.6.

### 2.4. Random Forests

Random Forests is an ensemble learning method used for classification and regression prediction [51,52]. The RF algorithm creates multiple decision trees that are defined with random feature selection (this process is also known as feature bootstrap aggregation or feature bagging). This is one of the strengths of the RF algorithm since with increased randomness of used features, the created decision trees have low variance and thus model overfitting is less likely to be a problem during the prediction process since there exists a de-correlation of each randomly constructed decision tree.

It was empirically shown that the RF algorithm outperforms the DT algorithm on multiple problems [53]. The generally most important RF algorithm training parameter is the used number of trees as the greater the number of trees is set, a more robust prediction will be achieved. The RF prediction process means that each randomly constructed decision tree creates its own prediction with the final decision or result being the most occurring one or rather the one with the most votes.

In the work by [54] the RF algorithm was compared other machine learning algorithms such as Logistic Regression, k-Nearest Neighbors, Support Vector Machine, Naive Bayes on a data classification problem regarding disease prediction and it showed better performance in accuracy. A recent review of RF algorithm applications specifically in the water resources applications filed is given by [55] where it was shown that is being increasingly utilized to build surrogate models. RF was used to enhance the low cost sensors performance for the purposes of air quality monitoring by [56] as the model’s prediction results were satisfactory when compared to empirical models. Additionally, [57] used RF in conjunction with remote sensing techniques for the purpose of dust source detection and mapping. It outperformed other machine learning algorithms such as Weights of Evidence (WOE) and Frequency Ratio (FR).

The RF implementation in the Python machine learning module scikit-learn 0.21.3. [43] was utilized for training the machine learning model for both algorithmic frameworks. The number of trees (the number of estimators) used for all RF models training in the algorithmic process was 250 while all of the other parameters were set as default. These settings were tuned with a grid search calibration of the RF model.

The RandomForestClassifier and RandomForestRegressor function from the scikit-learn 0.21.3 module ensemble were used for classification and regression, respectively. For both functions the argument used was the number of estimators or trees. After object creation the functions fit and predict were used to train the RF model and predict the values of interest.

### 2.5. Stochastic Optimization Algorithms

#### 2.5.1. Fireworks Algorithm

The fireworks optimization algorithm (FWA) is one of the investigated stochastic optimization algorithms in this study. It is a swarm intelligence algorithm generally used for optimization of complex goal functions and it is inspired by the process of fireworks explosions [58]. Ref. [59] created two frameworks for EEG signal data optimization which incorporated the single objective and multi objective FWA. In the work by [60] FWA was coupled with Evolutionary Computation for the purpose of classification and clustering on several different data sets. It was shown that it outperforms Particle Swarm Optimization for the same tasks.

The algorithm procedure includes randomly initializing a set of *n* fireworks for objective function evaluation with each of the *n* fireworks performing a local search through the search space. After each explosion a total number of explosion sparks $m_{1}$ are generated and the location of each explosion spark is obtained and evaluated. Better fireworks (in terms of fitness) will generate a greater number of sparks $m_{1}$ with a smaller amplitude of explosion while the ones with worse fitness values will contain a smaller number of sparks with a larger explosion amplitude. Additionally, a total of $m_{2}$ Gaussian mutated sparks are generated in order to increase diversity of the sparks. Every new generation of fireworks is constructed based on the fitness value, and both explosion sparks and the Gaussian mutated sparks. The FWA implementation in the python numerical optimization module indago 0.1.2 was used [44].

The indago 0.1.2. function evaluation_function was used to define the name of the optimization function, while the functions dimensions, iterations, lb, ub and params were used to define the number of optimization variables, iterations, lower bound, upper bound of the optimization problem and the optimization algorithm specific parameters, respectively. The run function was used to start the optimization loop while *f* and *X* were minimum fitness and optimization variables at minimum fitness functions, respectively.

#### 2.5.2. Particle Swarm Optimization

The second stochastic algorithm used in the preliminary analysis is the Particle Swarm Optimization (PSO) algorithm. PSO is a swarm intelligence algorithm inspired by the movement of birds [61]. A recent overview of the developments of PSO is given by [62]. PSO was used in the work by [63] for identifying the unknown groundwater contaminant sources as a part of the simulation-optimization procedure. In Ma et al. [64] PSO was used for gas emission source identification and compared with the firefly algorithm and Ant Colony Optimization algorithm. It was found that all of the three algorithms perform similarly in terms of estimating the source parameter but with PSO being computationally superior. In [65] PSO was used for the purpose of optimization of hydraulic demands of a water distribution network.

The particles which form a swarm with size *s* move through the objective search space with an inertia and are constantly both being attracted to the best position they have individually found and the best position determined by any other particle in the swarm. The parameters that influence the movement of each particle include the inertia factor *w*, cognitive and social factors $c_{1}$ and $c_{2}$. The languid particle dynamics modification [66] was used which involves setting the inertia of a particle to zero if it is not moving in the direction of better fitness. This modification was used as it proved beneficial to the standard PSO algorithm on the problem of water distribution pipe network routing [67]. The PSO implementation in the python numerical optimization module indago 0.1.2. was also used. The same indago functions were used as the one presented in the previous subsection but with different algorithm specific parameters set with the function params since PSO was used.

#### 2.5.3. Genetic Algorithms

The last examined stochastic algorithm is the genetic algorithm (GA) [68]. GA has been widely used in previous studies for the water network distribution contamination source detection problem [14,16]. A recent review of GA with a focus on the crossover and mutation rate choice was made by [69]. Recently, in the work by [70], GA was used to optimize a novel real-time control system for mitigation of sewer flooding and in [71] GA is implemented in an algorithmic procedure to investigate the wastewater seepage appearance in a semiarid urban environment.

GA is an algorithm inspired by the evolutionary process. A population (with size *p*) is formed by a set of individuals which are improved with each generation *g*. The formation of a new generation is based on the selection of the best performing individuals which is determined by their fitness value, the crossover parameter $c_{r}$ and the mutation *m*. The python module for multiobjective optimization pymoo 0.4.2. was used. The details and review of the specifics of the Python module pymoo can be found in the paper by the original authors [45].

The pymoo function FunctionalProblem was used to define the whole optimization function which takes in as function arguments the number of optimization variables, the name of the predefined goal function and the lower and upper bounds of the optimization problem. The function SingleObjectiveDefaultTermination was used to define the maximum number of generations as the stopping criterion. The mutation and crossover rates were defined with the functions get_mutation and get_crossover. The mutation rate, crossover rate, population size were used as arguments for the genetic algorithm function GA and finally, the optimization function, algorithm definition and the termination criterion were used as arguments for the pymoo minimize function.

#### 2.5.4. Preliminary Analysis

**Figure 8:**
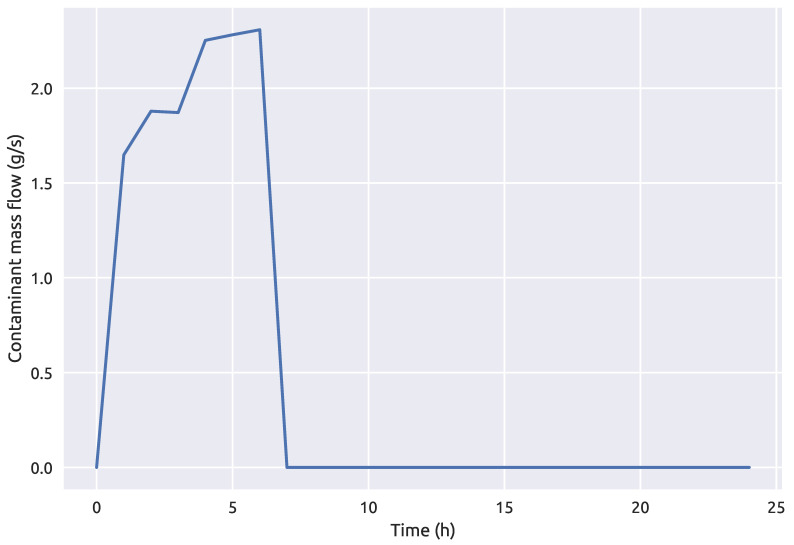
Average contaminant mass flow at the source node 261 over a 24 h period.

A preliminary analysis using the three different stochastic optimization algorithms was made and the best performing one was used for further investigation of the machine learning and simulation-optimization coupling framework 1. The preliminary analysis included finding the optimal solution or rather the values of $s_{t}$, $e_{t}$ and *c* for a known source node. The goal function was defined as in Equation (1) and the simulation-optimization procedure was done on the NET3 benchmark network with node 261 being the contamination source node. The true contamination event start time was 00:40 h, end time 06:30 and the injected contaminant concentration 78.5 mg/L. The optimization constraints for the start and end times were set as 00:00 h and 24:00 h (with a required condition that $s_{t}$ < $e_{t}$), while the injected contaminant concentration was bounded between 10 and. The contamination event parameters are summarized in Table 1 and the average contaminant mass flow which enters the node 261 can be observed in Figure 8. The maximum percentage of contamination mass thorough the time interval with relation to the total mass which passes through the true source node is 0.0078% for the proposed contamination event.

A parameter tuning process was also done for all of the three investigated algorithms through a grid search process. For each parameter combination of the three algorithms, a total of 100 repeated runs were made due to their stochastic nature. The performance was measured as the number of successful runs (cases in which all of the three optimization variables were predicted correctly with a fitness value below 0.02) out of 100 and the average time per run. A total of 256 parameter combinations were examined for each algorithm. The FWA varied values were iterations *i*, *n*, $m_{1}$ and $m_{2}$. For PSO the varied values were *i*, $c_{1}$, $c_{2}$ and the swarm size *s* and the GA varied values were *g*, *p*, $c_{r}$ and *m*. Summary of the results can be seen in Table 2. Out of the three algorithms, FWA has the best performance in terms of successful runs and the average time per run for the given parameters. The average value of the goal function *f* can also be seen in Table 2 with the lowest being achieved by PSO. FWA was used for further investigation of the coupling process within the algorithmic framework 1.

### 2.6. Deterministic Optimization Algorithm

#### Mesh Adaptive Direct Search

Mesh Adaptive Direct Search (MADS) was used as the deterministic optimization algorithm within the algorithmic framework 2 which includes the machine learning regression prediction model. MADS has not previously been used in research regarding water resources. In the recent work by [72] MADS optimization was used for the purposes autonomous vehicles control and in [73] it was successfully used to optimize the Gas-Lift procedure for maximizing the production of hydrocarbons from heavy oil and offshore reservoirs.

MADS is adequate for this kind of coupling as it must have an initial search condition which in this case is obtained from the RF regression (values of $s_{t}$, $e_{t}$ and *c*). MADS is an iterative method which includes creating a search space mesh for the optimization process [74]. The objective fitness search was achieved by mesh refinement within two essential steps—poll and search. The search step evaluates mesh points, and if progress of the fitness value is not achieved, a poll step performs a local search near the current best solution. If both steps do not find a better fitness value, the search space mesh is refined. The MADS implementation in the black box optimization open source software NOMAD 4.0 (developed at Polytechnique Montreal, Montreal, Canada) [46] was used. The only parameter used for the MADS optimization process was the number of goal function (Equation (1)) evaluations.

The NOMAD python wrapper PyNomad was used to form the optimization loop. The main NOMAD function used for evaluation is the optimize function, which takes in as arguments the name of the optimization function; the initial search condition; lower optimization bound; upper bound; additional parameters BB_OUTPUT_TYPEOBJ which define the output type (in this case the value of the objective function); and MAX_BB_EVAL, which is the maximum number of evaluations.

## 3. Results and Discussion

### 3.1. Random Forest Classifier Prediction

For both algorithmic frameworks and benchmark water distribution networks it is necessary to predict the top contamination source candidate nodes with the RF classifier. It is a requirement that the model is built before being employed in the algorithmic procedure. The whole process of training and prediction was repeated as in the work by [39].

The RF model classifier for the NET3 network was trained with 70,000 perfect sensor measurements (as input features) and true source nodes as the output features. The training and testing procedure lasted for 37 s on one INTEL E7 CPU core and an accuracy (30,000 input features) of 99% that the true source node was in the top 10 of the potential contamination source nodes predicted by the RF classifier was achieved. The trained classifier is then used within both coupling frameworks when NET3 network was investigated.

The same training process was repeated for the Richmond water distribution network with fuzzy sensor measurements as input features. Since the size of the network is larger that NET3 (865 potential source nodes), a total of 1,050,000 inputs were used for the RF model training and an accuracy of 99% was achieved for the true contamination source node being in the top 60 of the predicted potential source nodes. The training and testing process lasted for 955 s on the same hardware. A summary of both RF classifiers is given in Table 3.

### 3.2. Algorithmic Framework 1 Results

In this subsection the results of the coupling framework summarized in Section 2.2 are presented for both benchmark water distribution networks. All runs were done on one INTEL E7 node (manufactured by Intel Corporation, Santa Clara, CA, USA) with 256 cores. The FWA optimization algorithm is used with the tuned parameters specified in Section 2.5 for both networks.

The same NET3 contamination event parameters as presented in Table 1 were used to examine the efficiency and the robustness of the framework. The framework was repeatedly run for 100 times and for all runs the true source node (node 261) was in the top 10 of the potential source nodes predicted by the RF classifier and it was found in 99 out of 100 times after the simulation-optimization process had finished. After only one run, the selected contamination source node was node 263 as it is located closely to the true source node 261. This is a common occurrence in the contamination source detection procedure as the problem is greatly multimodal.

**Figure 9:**
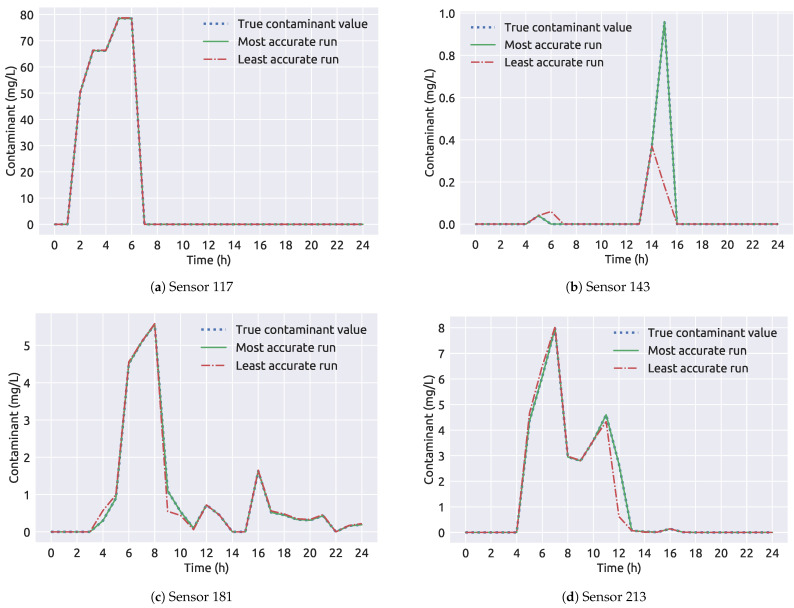
A comparison of sensor measurements through time for the NET3 benchmark network (framework 1).

A summary of framework 1 results for the NET3 network can be observed in Table 4 and it is shown that the algorithmic framework successfully determined the true source node contamination event parameters. The worst performing run is the one where the true source node was wrongly determined in terms while the best performing run has both the correct true source node and the best fitness. The values of average run correspond to the 99 runs when the true source node was node 261. In Figure 9 the sensor measurements of the contaminant through the 24 h period are shown. The least accurate result is the measurement for node 263 being the source node. It can be observed that the measurements between the most accurate and least accurate run differ greatly on sensor 143 (Figure 9b) while the measurement difference on other sensors in the network is minimal. The first coupling framework shows excellent convergence in a decent amount of time (average run was 142 s) for the NET3 benchmark network case with perfect sensor measurements.

**Figure 10:**
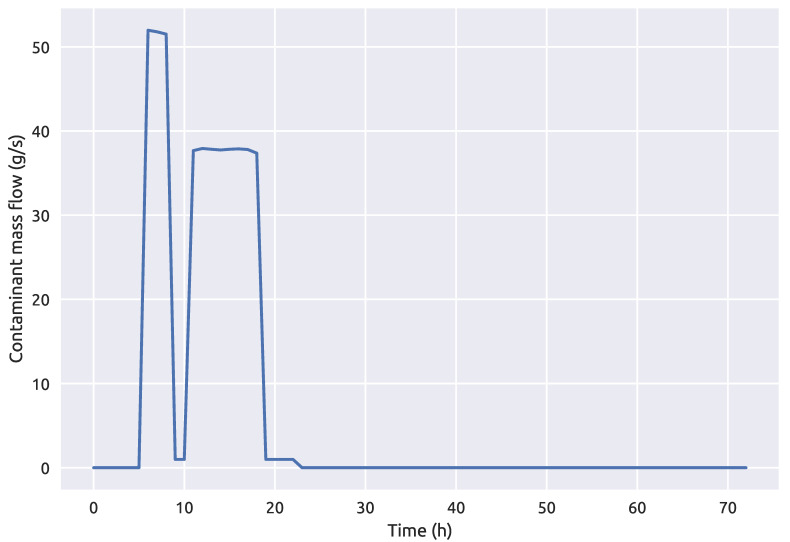
Average contaminant mass flow at the source node 251 for the Richmond network over a 72 h period.

The Richmond water distribution network contamination event parameters (start, end times and injected contaminant concentration with the network source node) can be seen in Table 5. The coupling framework 1 was also repeatedly run 100 times for the Richmond network with the fuzzy sensor measurements. The average contaminant mass flow at the true source node 251 can be seen in Figure 10 and the maximum percentage of contamination mass over the simulation time is 0.0938% for this benchmark network case.

**Figure 11:**
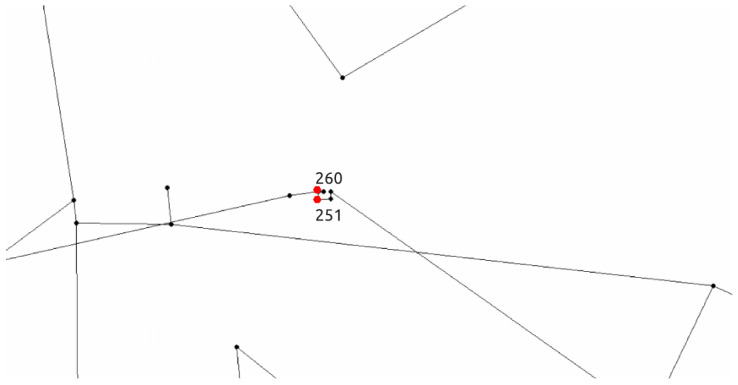
Network node locations of the two top candidate source nodes 251 and 260 (zoomed in part of the network).

For 89 of 100 runs, the true source node was a tie in terms of fitness between two network nodes (node 251 and node 260). This is expected since as sensor measurements are not perfect and the multimodal nature of this problem is enhanced. For the remaining 11 runs, node 251 was selected to be the true source node for 4 runs while node 260 for seven runs. Both nodes are located closely to each other in the Richmond water distribution network and this can be seen in Figure 11 and in Table 6, a summary of the results for the true source node 251 is presented.

Interestingly, all of the 100 runs have the final fitness value of 0.0. This can be explained due to the simplicity of the fuzzy sensor measurements and the before mentioned enhanced multimodality of this problem where many solutions are equally as good in terms of the computed fitness. Due to the equally good fitness of all runs the accuracy of the results was determined with a root mean square error analysis of the contamination event parameters. The least accurate run severely underestimates the end time of the contamination event while all other parameters are predicted with good accuracy.

In Table 7, the results for the source node 260 can be observed. Even though the source location is wrong, the results are also useful due to the proximity of node 260 to the true source node and the good average prediction in terms of contamination event initial values.

### 3.3. Algorithmic Framework 2 Results

The second algorithmic framework which includes the ML regression model was also investigated for both benchmark networks with NET3 contamination event scenario parameters presented in Table 1 and Richmond parameters in Table 5. The number of iterations of the MADS algorithm was set to 300 for both water distribution benchmark network contamination event investigation. Framework 2 was also run for 100 times for both benchmark networks, as framework 1.

For the NET3 network the total number of Monte Carlo generated input data for the ML regression analysis (the whole procedure shown in Figure 7) was 300,000, which means each node’s regression model for a top 10 potential source node list had an average of 30,000 inputs. An analysis for the RF regression predicted values (for true source node 261) for a 300,000 total inputs can be seen in Table 8. The RF regression is stable and robust as seen from the computed standard deviation of $s_{t}$, $e_{t}$ and *c*. The absolute error is the absolute distance from the contamination event parameters presented in Table 1 and it can be seen that the RF regression overestimates all of the three parameters, which is an additional proof that in order to reconstruct the whole contamination event scenario accurately, the simulation-optimization procedure is necessary.

**Figure 12:**
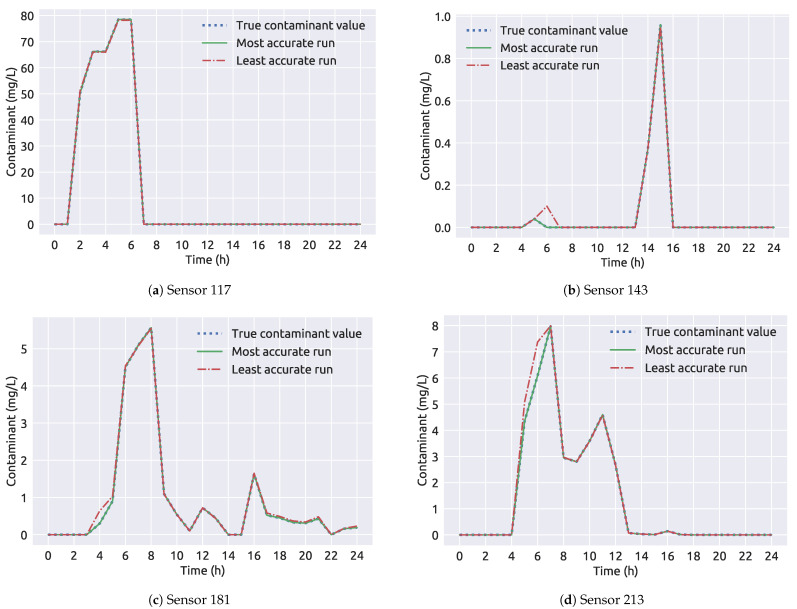
A comparison of sensor measurements through time for the NET3 benchmark network (framework 2).

In Table 9 the framework 2 results for the NET3 benchmark network are presented. The true source node 261 was selected as the source node for all 100 runs. The average run time is about 20 s shorter than the average run time for framework 1 (as seen in Table 4). While the end time and injected contaminant concentration predictions are of great accuracy, the start time is slightly underestimated with an absolute error of 0.09 h. In Figure 12 the comparison of sensor measurements for the most accurate and the least accurate run of framework 2 can be observed and even though that the start time of the least accurate run has a 00:40 h absolute error, the measurements show to be very similar over the time interval than those presented in Figure 9 for framework 1. This is due to the contamination source node being wrongly selected in the least accurate run from framework 1, while for framework 2, all of the 100 runs were correct in terms of the source location.

For the Richmond water distribution network, the true source node (node 251) was selected in all of the 100 runs. For 63 of the 100 runs the true source node was the only selected node of the framework while for the remaining 37 runs it was a tie between node 251 and 260. The total number of input data for the RF regression procedure was 785,000. The analysis of the RF predicted values (for true source node 251) can be seen in Table 10. The standard deviation for all three values is small which means that the RF prediction is robust while the absolute error is the biggest for the end time prediction. Table 11 and Table 12 show the results of 100 runs for the predicted source nodes. The average start time and the injected contaminant concentration are quite accurately predicted for the source node 251 while the end time of the contamination event scenario is slightly overestimated. Nonetheless, framework 2 with the MADS algorithm exhibits great robustness in determining the true source node as it was selected 63 out of 100 times as the only source node.

### 3.4. Framework Comparison

Both frameworks presented in this study have shown robustness and good accuracy in determining the contamination source node and parameters of the contamination event. Framework 1 has shown to be more accurate than framework 2 in determining the values of $s_{t}$, $e_{t}$ and *c* for both benchmark networks when the average values of 100 repeated runs are observed, however framework 2 has shown to obtain good results in less time. The greatest benefit of framework 2 which includes the RF regression model is that it is extremely robust in determining the true source node for the fuzzy sensor measurements benchmark example as it outperformed framework 1. In Table 13 the true source node detection comparison for the two presented frameworks is given.

## 4. Conclusions

In this study two algorithmic frameworks for water distribution network contamination event detection were presented. Both frameworks were tested on a small water distribution benchmark network with 92 potential sources with perfect sensor measurements and a bigger benchmark network with 865 potential sources which included fuzzy sensor measurements to examine the robustness of the frameworks.

The first algorithmic framework includes coupling a ML classification model based on the RF algorithm and a stochastic optimization algorithm. After a preliminary analysis and parameters calibration procedure on the smaller benchmark network, the fireworks algorithm showed to be superior to the Particle Swarm Optimization algorithm and the genetic algorithms which are the most popular optimization algorithms for the water network contamination source detection problem. The algorithmic framework with the Fireworks algorithm shows to work with good accuracy in predicting the start time, end time and injected contaminant concentration for both benchmark networks but lacks the robustness of predicting the true source node with fuzzy sensor measurements.

The second presented algorithmic framework has an added ML regression model for each of the potential source nodes generated by the RF classifier. The regression model is trained pre-generated data by Monte Carlo simulations in parallel. The framework was coupled with the Mesh Adaptive Direct Search algorithm which is extremely well suited for this procedure as it requires an initial search value which in this case is generated by the RF regression model. This framework showed to be robust and can predict with good accuracy the true source node when the contamination event incorporates fuzzy measurements.

The proposed methodology differs from other methods for contamination source node identification, as it combines the two more general approaches in a whole framework. Usually the simulation-optimization methods and data-driven machine learning based methods are uncoupled and used separately for the task of contamination source detection. With this approach, the strength of identifying the most probable source nodes via a machine learning algorithm is coupled with the strength of finding the start time, end time and injected contaminant concentration through simulation-optimization algorithms. The proposed methodology is computationally efficient since a search space reduction is achieved with the machine learning approach.

Hydraulic demand uncertainties of the water distribution networks should be included in future studies as they were not investigated with this framework, but as shown in [39], the RF classifier accuracy is slightly lowered when they are incorporated. In future studies other ML algorithms could be tested for the classification part of both algorithmic frameworks and the regression part of the second framework. Additionally, other optimization algorithms (stochastic and deterministic) could also be incorporated into both algorithmic frameworks and investigated.

## Figures and Tables

**Table 1:** NET3 network contamination event parameters.

Source Node	Start Time	End Time	Contaminant Concentration
261	00:40 h	06:30 h	78.5 mg/L

**Table 2:** Preliminary analysis of stochastic optimization algorithms—tuned parameters.

Algorithm	Successful Runs	Average Time per Run	Average *f*	Parameters
GA	100/100	121.5 s	0.015	*g*: 100, *p*: 80, $c_{r}$: 0.8, *m*: 0.1
PSO	98/100	178.6 s	0.003	*i*: 50, *s*: 100, $c_{1}$: 1, $c_{2}$: 1
FWA	100/100	64.13 s	0.009	*i*: 80, *n*: 5, $m_{1}$: 5, $m_{2}$: 10

**Table 3:** Trained RF classifier characteristics.

Network	Sensors	Inputs	Top Nodes	Accuracy	Time
NET3	Perfect	70,000	10	99%	37 s
Richmond	Fuzzy	105,000	60	99%	955 s

**Table 4:** Framework 1 results for the NET3 network.

Run	Source Node	Start Time	End Time	Contaminant Concentration	*f*	Time
Average	261	0:39 h	6:29 h	78.49 mg/L	0.024	142 s
Most accurate	261	0:40 h	6:30 h	78.5 mg/L	0.002	133 s
Least accurate	263	0:20 h	6:20 h	78.7 mg/L	0.436	117 s

**Table 5:** Richmond network contamination event parameters.

Source Node	Start Time	End Time	Contaminant Concentration
251	06:30 h	21:30 h	939.37 mg/L

**Table 6:** Framework 1 results for the Richmond network for the source node 251.

Run	Start Time	End Time	Contaminant Toncentration	*f*	Time
Average	6:30 h	21:00 h	940.6 mg/L	0.0	882 s
Most accurate	6:30 h	21:30 h	939.6 mg/L	0.0	852 s
Least accurate	6.30 h	11:40 h	943.9 mg/L	0.0	860 s

**Table 7:** Framework 1 results for the Richmond network for the source node 260.

Run	Start Time	End Time	Contaminant Concentration	*f*	Time
Average	6:30 h	20:09 h	915.6 mg/L	0.0	882 s
Most accurate	6:30 h	21:30 h	924.1 mg/L	0.0	843 s
Least accurate	6.30 h	14:50 h	908.4 mg/L	0.0	1002 s

**Table 8:** NET3 RF regression average values and standard deviations for 100 runs with absolute error.

	Average Prediction	Standard Deviation	Absolute Error
Start time	1:58 h	0:079 h	1:18 h
End time	6:56 h	0:15 h	0:16 h
Contaminant concentration	108.87 mg/L	5.17 mg/L	30.38 mg/L

**Table 9:** Framework 2 results for the NET3 network.

Run	Source Node	Start Time	End Time	Contaminant Concentration	*f*	Time
Average	261	0:31 h	6:30 h	78.48 mg/L	0.033	126 s
Most accurate	261	0:40 h	6:30 h	78.5 mg/L	0.002	124 s
Least accurate	261	0:00 h	6:30 h	78.2 mg/L	0.19	109 s

**Table 10:** Richmond RF regression average values and standard deviations for 100 runs with an absolute error.

	Average Prediction	Standard Deviation	Absolute Error
Start time	6:53 h	0:015 h	0:28 h
End time	17:57 h	0:049 h	3:73 h
Contaminant concentration	933.97 mg/L	0.96 mg/L	5.4 mg/L

**Table 11:** Framework 2 results for the Richmond network for the source node 251.

Run	Start Time	End Time	Contaminant Concentration	*f*	Time
Average	6:30 h	22:22 h	942.2 mg/L	0.0	632.7 s
Most accurate	6:30 h	21:30 h	939.7 mg/L	0.0	602 s
Least accurate	6.30 h	22:20 h	944.3 mg/L	0.0	591 s

**Table 12:** Framework 2 results for the Richmond network for the source node 260.

Run	Start Time	End Time	Contaminant Concentration	*f*	Time
Average	6:30 h	15:73 h	917.8 mg/L	0.0	632.7 s
Most accurate	6:30 h	17:40 h	924.3 mg/L	0.0	570 s
Least accurate	6.30 h	15:40 h	908.5 mg/L	0.0	596 s

**Table 13:** A comparison of frameworks 1 and 2 in determining the true source node for both network benchmarks.

Framework	Network	Runs	True Source	False Source	Tie
1	NET3	100	99	1	0
2	NET3	100	100	0	0
1	Richmond	100	4	7	89
2	Richmond	100	63	0	37

## Data Availability

Data available on request from the authors.

## Abbreviations

The following abbreviations are used in this manuscript:

ANN	Artificial Neural Network
CNN	Convolutional Neural Network
DT	Decision Tree
GA	Genetic Algorithms
LVQNN	Learning Vector Quantization Neural Network
MC	Monte Carlo
ML	Machine Learning
NM	Nelder-Mead
NSGA-II	Non-dominated Sorting Genetic Algorithm-II
PM	Powell’s method
PNN	Probabilistic Neural Networks
PSO	Particle Swam Optimization
PSVM	Probabilistic Support Vector Machines
RF	Random Forests
